# Large-scale energy infrastructure projects and thoroughbred stud farms: equine welfare, evidence gaps and planning considerations

**DOI:** 10.1186/s13620-026-00367-x

**Published:** 2026-10-23

**Authors:** W. M. Bayly, D. R. Hodgson, D. P. Leadon

**Affiliations:** 1 Department of Veterinary Clinical Science, Washington State University, Pullman, Washington USA; 2 Large Animal Clinical Sciences, 205 Duck Pond Drive, Virginia-Maryland College of Veterinary Medicine (0442), Blacksburg, VA 24061 USA; 3 Irish Equine Centre, Johnstown, County Kildare Ireland

**Keywords:** Equine welfare, Renewable energy, Thoroughbred breeding, Stud farms, Noise sensitivity, Planning policy, Ireland

## Abstract

Industrial-scale renewable energy developments i.e. solar photovoltaic (PV) installations and wind energy facilities, form a central component of Ireland’s national decarbonisation strategy. As these infrastructures expand, they are increasingly being proposed or developed within rural regions that also contain high densities of Thoroughbred breeding establishments. This creates a potential interface between national energy policy, rural land use, equine welfare and the operational requirements of the Thoroughbred industry which is of national and international importance.

Direct empirical evidence examining the effects of large-scale renewable energy developments on Thoroughbred mares, foals and youngstock on adjacent stud farms remains limited. However, established evidence from equine sensory physiology, behavioural science, veterinary medicine and environmental studies demonstrates that horses are highly responsive to noise, visual stimuli, airborne particulates and sudden environmental disturbance. These findings provide biologically plausible pathways through which construction and, in some circumstances, operation of large-scale energy infrastructure may adversely affect equine behaviour, health and welfare.

Current Irish planning legislation does not specifically require equine welfare considerations to be incorporated into decision-making for developments adjacent to breeding establishments. This commentary argues that the absence of direct equine-specific evidence should not be interpreted as evidence of absence of risk. Rather, it supports a proportionate and precautionary approach incorporating equine-specific assessment, independent veterinary input, appropriate mitigation and site-specific planning where major renewable energy infrastructure is proposed in close proximity to stud farms.

## Introduction

Irelands commitment to renewable energy generation is necessary and important. However, major infrastructure development also creates a requirement to consider how new land uses interact with established rural activities, particularly where these activities involve animals whose management, behaviour and welfare are closely linked to environmental conditions.

This issue is particularly relevant in counties such as Meath, Kildare and Tipperary, which have both high densities of Thoroughbred breeding establishments and increasing levels of renewable energy development. Ten of the 20 largest solar farms, some extending to 700 acres or more, are expected to be located within these principal breeding counties, with respective totals of 17, 10 and 14 large-scale individual sites in each county [1].

Applications have already been made, and in several instances approved, for developments adjacent to stud farms. These developments are substantial and include the erection of 250,000 solar panels mounted on pile driven supports which can be 10 feet high and 20 feet wide arranged in rows, with 50 or more electrical transformer units surrounded by 8 feet high security fencing with CCTV cameras. They require internal roadways and access from public roadways to enable up to 2,400 HGV movements to enable their construction. Many bloodstock industry objections have been raised to these applications but planning authorities have reached differing conclusions when considering potential impacts on bloodstock enterprises. Although bloodstock issues were recognized as a substantive planning issue for refusal in one instance those same concerns have been discounted in others [2].

The purpose of this commentary is to examine whether current planning frameworks sufficiently recognise the particular behavioural, sensory, health and management characteristics of horses when large-scale energy infrastructure is proposed in proximity to Thoroughbred breeding establishments.

## The particular context of thoroughbred stud farms

Breed-related differences in emotional reactivity and behavioural responses have been reported in horses, and Thoroughbreds are generally regarded as among the more behaviourally reactive breeds used in equestrian activities [3–5] .

A Thoroughbred breeding establishment presents a different management context from land on which mature riding or leisure horses are ordinarily kept. Pregnant mares, mares with foals at foot, neonatal foals, yearlings and youngstock spend significant periods at pasture and routinely move between paddocks, yards, foaling areas and veterinary or handling facilities. Mares are naturally protective of their foals and exhibit heightened responsiveness to environmental disturbance during the foal-rearing period [6, 7].

These characteristics indicate that breeding establishments require particular consideration where nearby construction or operational activity introduces novel, irregular or intense environmental stimuli.

## Current planning context

The European Union (Planning and Development – Renewable Energy) Regulations 2025 introduced new definitions and criteria for renewable energy projects [8]. The Regulations are intended to facilitate the development of renewable energy infrastructure but do not currently contain specific provisions relating to equine welfare.

Ireland’s Animal Health and Welfare Act 2013 [9] provides a statutory framework for animal protection, while the Animal Welfare Strategy 2021–2025 [10] reaffirmed the Government’s commitment to continuing improvement in animal welfare.

However, animal welfare legislation and planning legislation remain largely separate in their practical application. As noted in recent legal scholarship:

“The general approach of planning authorities in the Republic of Ireland is not to have regard to animal welfare matters at the planning stage” [11].

The scale and form of contemporary renewable energy infrastructure were not significant considerations when the Planning and Development Act 2000 [12] was enacted. This raises a legitimate question as to whether current planning processes adequately address animal welfare where development is proposed immediately adjacent to intensive equine breeding operations.

Planning frameworks should reasonably require consideration of equine-specific factors where the scale, proximity or nature of a proposed development makes them relevant.

Such consideration should include environmental impact assessments (EIA’s) veterinary-reviewed Animal Welfare Impact Statements, independent expert review and appropriate monitoring where substantial infrastructure is proposed close to breeding establishments.

## Equine sensory and behavioural sensitivity

### Noise

Noise-related anxiety and startle responses are well recognised in horses [13]. As a prey species, horses possess highly developed auditory capabilities that support rapid detection of potential environmental threats.

Irregular, unpredictable or sudden noise is particularly relevant because it may trigger hypervigilance, startle responses and flight behaviour, even when absolute sound intensity is not extreme [14]. An illustrative example occurred during the widely reported Household Cavalry incident in London in April 2024, when horses were startled by construction rubble being dropped during nearby building works. Several horses bolted through central London, resulting in injuries to horses and riders [15]. This incident demonstrates the potential behavioural consequences of sudden, unfamiliar construction-related noise even in highly trained military horses.

Reported normal agricultural grazing background noise levels relevant to stud farm environments range from approximately 25 to 35 dB, while 38 dB has been cited as a baseline for stabled horses [16]. Noise associated with a motorway at a distance of approximately 500 m, at levels of ≥ 54 dB, has been shown to cause changes in equine behaviour and physiology [17]. This is an important threshold because it is a recent, peer reviewed pasture based study.

Conference proceedings describing noise exposure at the Melbourne Cup which reported noise levels of up to 76 dBA and 85 dBA in an environment containing approximately 120,000 people [18] have been cited by developers in their planning applications. These observations relate to mature, trained and habituated racehorses within a racecourse environment. The author of the original report has stated that he did not intend the findings to be used to try to establish acceptable thresholds for stud farms [19]. Such findings should not be interpreted as a proxy for acceptable noise exposure on breeding farms. The physiological and behavioural context of a mature racehorse habituated to racecourse activity differs substantially from that of a mare with a young foal at pasture in a normally quiet rural environment.

Similarly, another study quoted by developers examined the effects of simulated jet fighter low level flying aircraft noise of up to 113.6 dB on stabled Thoroughbred mares [20]. Although this study provided useful information regarding equine responses to intense auditory stimuli which sought to replicate that arising from low flying jet fighter aircraft, important limitations arise when attempting to extrapolate its findings to breeding farms.

Experimental protocols deliberately exposing neonatal foals, mares with foals or untrained youngstock to potentially harmful levels of environmental noise would present significant ethical and welfare challenges.

### Construction noise and pile driving

Pile driving, which is required for solar-panel mounting systems, is recognised as one of the loudest forms of construction activity. Reported pile driving noise levels are approximately 101 dB at 15 m and may approach 120 dB in close proximity to the source [21] and this noise is repeated tens of thousands of times in large scale solar developments, which may contain 230,000 or more solar panels.

For this reason, developments involving substantial pile-driving activity in close proximity to breeding paddocks, foaling areas or youngstock warrant specific acoustic assessment and mitigation, rather than reliance on general human or residential noise thresholds alone.

## Visual disturbance

Horses possess a highly specialised visual system adapted for early detection of movement and potential threat.

Their visual field exceeds 300°, and they are particularly sensitive to movement within the peripheral visual field. Construction vehicle movements and their indicator and reversing warning lights, sudden reflections, glare, intermittent shadows, blade movement, solar-panel reflections or changes in light and motion may therefore be perceived differently by horses than by humans [22]. There is currently insufficient direct evidence to conclude that such effects from renewable energy installations during construction (solar) and operation (wind turbines) routinely produce adverse welfare outcomes in horses. Nevertheless, established equine sensory physiology provides a biologically plausible basis for considering visual disturbance where large-scale infrastructure is located immediately adjacent to paddocks or areas containing mares, foals or youngstock.

Visual assessment should therefore form an essential part of site-specific equine welfare consideration, particularly where moving, reflective or intermittent visual stimuli are likely to occur.

## Dust and air quality

Respiratory health is an important welfare consideration in horses, and exposure to airborne particulate matter is well established as a risk factor for inflammatory airway disease. Construction associated with large-scale solar or wind infrastructure requires excavation, heavy vehicle movement, disturbed topsoil, access-road development and other activities capable of generating particulate matter/dust. Dust exposure may contribute to airway hyperresponsiveness, coughing, reduced respiratory function and exercise intolerance and may also reduce mucociliary clearance and increase susceptibility to respiratory infection [23]. The degree of risk will vary according to particle size, composition, prevailing wind, moisture levels, distance and duration of exposure. Organic material, silica and other particles associated with disturbed soil may add to the overall exposure burden. The available evidence does not establish that construction of renewable energy infrastructure necessarily produces clinically significant respiratory disease in horses on neighbouring farms. It does, however, demonstrate a credible exposure pathway that warrants consideration where substantial dust-generating activity is proposed in close proximity to breeding animals where young foals may be particularly vulnerable to respiratory irritation, and subclinical airway inflammation which may have consequences for health, development and welfare [24]. Planning considerations should therefore also include baseline air-quality assessment, dust monitoring, effective suppression strategies, traffic management and site-specific separation distances.

## Fire risk and emergency planning

Fire events involving solar PV installations or electrical infrastructure appear to be relatively infrequent. However, where fires do occur, their consequences may be significant. Potential hazards include inverter or transformer faults, cable failures and electrical arc events. These may generate heat, flames and smoke. Horses may react strongly to smoke, unusual odours, flames and emergency activity. Smoke exposure may also increase respiratory effort and cause distress [25]. The challenge is particularly relevant on breeding farms because horses generally require human assistance during emergency evacuation, while mares with foals may be more difficult to move rapidly and safely under stressful conditions. Renewable infrastructure may also alter access routes or introduce fencing, substations, cabling or other physical infrastructure that could affect emergency movement or fire-service access. Accordingly, although fire risk is low-frequency, it is potentially high-consequence and should be incorporated into site-specific emergency planning where renewable energy installations are proposed immediately adjacent to equine premises.

## Construction and operational impacts should be distinguished

An important consideration in assessing renewable energy developments is the distinction between temporary construction-related exposure and longer-term operational exposure. Construction exposures are often intermittent, unpredictable and relatively intense. Operational exposures are different and may include low-level mechanical noise, transformer or inverter activity, maintenance traffic, turbine movement, changes in access and fencing. Evidence relating to one phase should not automatically be extrapolated to the other.

Equine-specific planning assessments should therefore distinguish clearly between construction and operational risks and identify mitigation appropriate to each.

## Mitigation strategies and their limitations

Several mitigation measures may be considered where major infrastructure is proposed in proximity to stud farms. These may include temporary relocation, increased stabling, alteration of foaling or turnout areas, construction scheduling, acoustic barriers, dust suppression, emergency planning and appropriate separation distances.

Relocation to other paddocks may reduce exposure in some circumstances but may not be feasible where breeding farms have limited alternative land or where established foaling and youngstock areas cannot readily be moved.

Increasing stabling time may similarly reduce exposure to some external stimuli but may create other welfare and management concerns. Prolonged confinement limits normal social interaction and locomotor behaviour and does not represent a proportionate long-term response to external development activity [26, 27].

Sedation may reduce acute anxiety temporarily but carries implications for coordination, vigilance and normal behaviour and is not suitable for managing recurrent environmental disturbance. Routine or repeated pharmacological sedation contravenes animal welfare and thus cannot reasonably be considered a sustainable mitigation strategy.

Acoustic barriers may provide useful attenuation where correctly designed and positioned. Timber acoustic fencing, absorptive composites, concrete structures and proprietary acoustic-wall systems may reduce noise at nearby receptors. Their effectiveness depends on barrier density, height, distance from the source and whether the barrier interrupts the line of sight between source and receiver. Such barriers may be less effective against irregular, percussive or low-frequency noise [28].

Where barriers or other mitigation measures are required specifically because of a proposed development, the cost of those measures should appropriately form part of the development rather than being transferred to the adjoining stud farms.

Construction scheduling may reduce cumulative exposure but cannot eliminate sudden noise, vibration or machinery events.

Mitigation should therefore not be regarded as a substitute for appropriate siting. In some circumstances, separation between a development and sensitive equine areas may represent the most effective means of reducing risk. Separation measures can also include re-location of stock from the affected farm for the duration of construction.

## Setbacks and separation distances

Setback distances / physical separation between industrial scale renewable infrastructures and sensitive receptors, (usually houses) have been introduced or proposed for wind energy developments in several jurisdictions. Examples include proposals or provisions in France, the United Kingdom, Kentucky and Bavaria, including separation distances ranging from approximately 1,000 ft to 2,000 m or more depending on jurisdiction and turbine characteristics [29].

However, these approaches arise from differing legal, environmental and planning contexts and have not necessarily been developed specifically for the protection of horses. They should therefore not be interpreted as demonstrating a universal equine-specific safe distance.

A more defensible approach would be to determine separation distances on a site-specific basis that reflects the characteristics of the proposed development and the sensitivity of nearby receptors. Setback distances developed for wind turbines cannot automatically be transferred to solar photovoltaic (PV) developments because the nature, intensity and duration of potential exposures differ. Inverters and transformers generate continuous operational noise that may contribute to the local acoustic environment and, potentially, to chronic acoustic disturbance in nearby horses. Locating these components away from sensitive areas of stud farms, particularly foaling units, may help minimise unnecessary exposure during critical reproductive and neonatal periods.

Accordingly, risk assessment should focus on the specific characteristics of the proposed development, the horses exposed, and the pathways through which disturbance may occur, rather than relying solely on arbitrary separation distances.

## Evidence gaps

Perhaps the most important limitation in this area is the lack of direct empirical research investigating horses maintained on breeding establishments adjacent to large-scale renewable energy infrastructure. Priority areas for future research should include prospective monitoring of equine behaviour and physiology before, during and after construction; measurement of noise and vibration in breeding-farm environments; investigation of visual stimuli and startle responses; particulate monitoring; and longitudinal assessment of mares, foals and youngstock exposed to nearby infrastructure development.

Such research would allow future planning decisions to move from precautionary assessment toward more robust evidence-based thresholds.

## Towards an equine-sensitive planning framework

The current evidence supports legislative recognition of breeding establishments as a sensitive receptor where major infrastructure is proposed in close proximity.

A proportionate planning framework could include: Table 1.

**Table 1:** A framework for planning considerations

Issue	Suggested assessment
Noise	Baseline and construction monitoring
Dust	IAQM-based controls
Welfare	Veterinary Welfare Impact Statement
Fire	Emergency planning
Visual disturbance	Site-specific assessment

Such an approach would provide a structured mechanism through which renewable energy objectives and equine welfare considerations could be addressed together.

## Conclusions

Ireland’s transition toward renewable energy is both necessary and likely to result in increasing interaction between large-scale energy infrastructure and existing rural industries. The central policy challenge is not to determine whether renewable energy development and Thoroughbred breeding are inherently incompatible. Rather, it is to ensure that developments are planned in a way that recognises equine breeding establishments as important and sensitive receptors and that credible risks are assessed fairly, consistently and proportionately before construction begins and are monitored thereafter. The current absence of direct equine-specific evidence should therefore prompt further research rather than regulatory inaction.

## Data Availability

No datasets were generated or analysed during the current study.
